# Analysis of Synaptic Microcircuits in the Mushroom Bodies of the Honeybee

**DOI:** 10.3390/insects11010043

**Published:** 2020-01-07

**Authors:** Claudia Groh, Wolfgang Rössler

**Affiliations:** Behavioral Physiology & Sociobiology, Biozentrum, University of Würzburg, Am Hubland, 97074 Würzburg, Germany

**Keywords:** mushroom body, microglomeruli, projection neurons, Kenyon cells, dendritic specializations, structural synaptic plasticity, behavioral plasticity, vision, olfaction

## Abstract

Mushroom bodies (MBs) are multisensory integration centers in the insect brain involved in learning and memory formation. In the honeybee, the main sensory input region (calyx) of MBs is comparatively large and receives input from mainly olfactory and visual senses, but also from gustatory/tactile modalities. Behavioral plasticity following differential brood care, changes in sensory exposure or the formation of associative long-term memory (LTM) was shown to be associated with structural plasticity in synaptic microcircuits (microglomeruli) within olfactory and visual compartments of the MB calyx. In the same line, physiological studies have demonstrated that MB-calyx microcircuits change response properties after associative learning. The aim of this review is to provide an update and synthesis of recent research on the plasticity of microcircuits in the MB calyx of the honeybee, specifically looking at the synaptic connectivity between sensory projection neurons (PNs) and MB intrinsic neurons (Kenyon cells). We focus on the honeybee as a favorable experimental insect for studying neuronal mechanisms underlying complex social behavior, but also compare it with other insect species for certain aspects. This review concludes by highlighting open questions and promising routes for future research aimed at understanding the causal relationships between neuronal and behavioral plasticity in this charismatic social insect.

## 1. Introduction

The honeybee represents a powerful experimental model for investigating the changes in synaptic circuits that occur in the brain during adult behavioral development and learning in an insect that has a complex social life. The ability of nervous systems to form and maintain neuronal connections in response to internal and external influences, and to modify and reorganize them during different life stages, represents a most fascinating area of the behavioral neurosciences [1,2,3].

Honeybees emerge into adult life inside a colony of thousands of individuals inside a more or less dark hive providing plenty of sensory stimuli related to social interactions—especially olfactory, gustatory or tactile stimuli. Following an age-related change in the spectrum of behaviors (age polyethism), young worker bees first perform several tasks inside the dark hive for about three weeks before starting to forage outdoors under bright sunlight (e.g., [4]; for review see [5]). The drastic interior–exterior transition exposes bees to completely new sensory environments and puts different demands on new tasks like spatial orientation or exploring and memorizing profitable food sources in a highly variable visual and olfactory environment [6]. Mushroom bodies (MBs), prominent centers in the honeybee brain, are neuronal substrates for multisensory integration, learning and memory formation [7,8,9]. They are viewed as an experience-dependent re-coding device transforming a multidimensional sensory input into a low dimensional code of value-based information, which enables the insects to quickly adapt to or learn even complex changes in their environment [10].

Structural analyses of microcircuits at the main MB input (MB-calyx microglomeruli, MG) have been performed along the transition from inside the dark hive to outdoor foraging (interior–exterior transition) and correlated with external and internal variables such as conditions that occur before adult emergence during brood care and during age- and task-related adult behavioral maturation [11,12,13,14,15,16,17]. Learning experiments on foraging adult bees have revealed that the formation of stable olfactory long-term memory (LTM) leads to structural neuronal plasticity of microcircuits in the MB calyces [18]. Similar results were obtained in leaf cutting ants after the formation of aversive olfactory LTM, resulting in the long-term rejection of unsuitable plant materials [19]. The results show that non-associative and associative sensory inputs trigger different forms of structural synaptic plasticity in MB-calyx microcircuits that are accompanied by distinct changes in behavior [3,20]. Functional imaging studies of physiological parameters have revealed that response properties of MB-calyx microcircuits change after sensory exposure and learning, giving further support to the behavioral relevance of changes in the wiring of MB-calyx microcircuits [21,22,23]. The relevance of structural plasticity in MB-calyx microcircuits has also been shown in the fruit fly, *Drosophila melanogaster*, by linking structural synaptic changes with physiological and molecular processes [24,25].

How are the numerous parallel synaptic microcircuits in the MB calyx of the honeybee organized, and how do early sensory exposure, learning and the formation of associative LTM change the synaptic connectivity and function of these circuits? This review highlights and synthesizes recent research on these issues with a main focus on the honeybee. The goal is to stimulate future multidisciplinary approaches aimed at understanding causal relationships between neuronal and behavioral plasticity in a social insect.

## 2. Honeybee Mushroom Bodies

**Figure 1:**
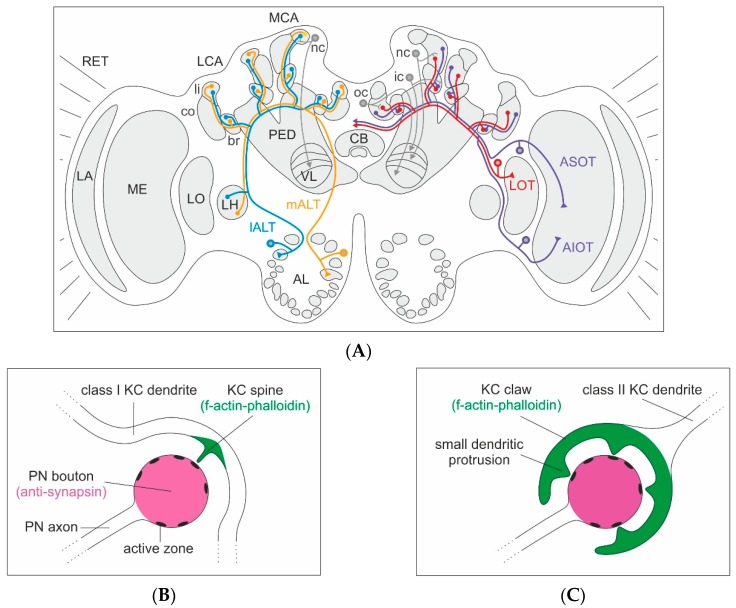
Olfactory and visual innervation of the medial (MCA) and lateral calyx (LCA) of the mushroom bodies (MBs), and the classification of Kenyon cells (KCs) in the honeybee brain. (**A**) Left brain hemisphere: Brain overview with innervation from projection neurons (PNs) of the medial (m-ALT, orange) and lateral (l-ALT, blue) antennal-lobe tracts to a spiny (class I) non-compact (nc) KC from the lip (li) of the MCA, with axonal projections to the lip layer of the vertical lobe (VL). Right brain hemisphere: PNs from the medulla (ME) project via the anterior superior (ASOT, purple) and anterior inferior optic tracts (AIOT, purple), as well as the lobula (LO) PNs via the lobular tract (LOT, red) (optic lobe tracts after [35]). Connections in the collar (co) with a spiny nc KC in the MCA with axonal projections to the visual layer of the VL, a spiny (class I) inner compact (ic) KC in the basal ring (br) projecting to the upper-most layer of the VL and a clawed (class II) outer compact (oc) KC projecting to the lower-most (gamma) layer of the VL. The KC axonal projections in the VL layers reflect the concentric organization of their dendrites in the MB calyx (KC projections after [28]). (**B**) Organization of an individual microglomerulus innervated by a class I KC. Schematic drawing of an individual PN synaptic bouton and innervation by a single spiny class I KC dendrite with one spine-like protrusion forming a synaptic contact at one active zone of the PN bouton. Anti-synapsin labeling colored in magenta and f-actin-phalloidin staining in green. (**C**) Organization of an individual microglomerulus innervated by a class II KC. Schematic drawing of a single PN synaptic bouton innervated by an individual claw of a class II KC dendrite with multiple dendritic protrusions forming synaptic contacts with multiple active zones of the PN bouton. Further abbreviations: AL: antennal lobe, CB: central body, LA: lamina, LH: lateral horn, PED: peduncle, PN: projection neuron, RET: retina.

In honeybees, MBs comprise about 368,000 intrinsic neurons (Kenyon cells (KCs)), making up more than 40% of the total number of brain neurons (for a review see [3]; see also [26,27,28]). For comparison, in the fruit fly, *Drosophila melanogaster*, there are an estimated 4000 KCs comprising no more than 4% of brain neurons [29]. This suggests a clear shift in the role of the MBs between the two species. The MBs of fruit flies have a more or less uniform MB calyx that predominantly functions as a higher olfactory association center [29,30]. Small MB calyces have also been found in sawflies (Symphyta), a group of plant-feeding basal Hymenoptera [31,32]. In the honeybee, the MB calyx receives prominent visual innervation in addition to olfactory input and, to some extent, projections from gustatory neurons, resulting in large, doubled, cup-shaped multimodal MB calyces (Figure 1A; see also [33,34]). Farris and Schulmeister [31] refer to the MB calyces in the brain of honeybees and other higher Hymenoptera as morphologically elaborate and speculate that the massive expansion of the MB calyx within the hymenopteran lineage was associated with the evolution of parasitoid lifestyles and the need for complex spatial orientation during food provisioning. They conclude that the expansion of MB calyces, most likely, was one pre-adaptation for the evolution of social lifestyles that require enhanced spatial orientation skills during central-place foraging, an important prerequisite for collective brood care in social insects.

In the honeybee, each of the four large MB calyces is anatomically subdivided into three distinct compartments—lip, collar and basal ring—receiving sensory input from olfactory, visual and both modalities, respectively (Figure 1A; see also [33,35,36]). Modality-specific MB-calyx compartments are characterized by a concentrically layered sensory input from projection neurons (PNs). The cortical and central input regions of the MB-calyx lip are innervated by ~400 olfactory PNs from the medial and ~500 from the lateral antennal lobe tracts (m- and l-ALT; Figure 1A; see also [36,37,38,39,40]; tract nomenclature after [41]). The collar is organized into an outer dense and inner loose synaptic region [11,13], receiving input from visual PNs emerging from the optic lobe medulla (ME) and lobula (LO) via three distinct tracts (Figure 1A; see also [35]). The medulla processes color information and presumably extracts motion information from the visual input, whereas the lobula conveys both color and motion information to the MB calyces [42,43]. The numbers of PNs in the visual tracts are not yet available. The concentric organization and segregation of sensory input in MB-calyx compartments is largely maintained at the level of the MB lobes, the major output region of the axons from different classes of KCs (Figure 1A; see also [28]). In the vertical lobe (VL) of the MB, axon terminals from KCs form distinct layers. KCs with dendrites in the basal ring terminate in the dorsal-most division and above the KCs, innervating the collar. The axons of KCs emerging from the lip terminate in a layer formed at about the midline of the VL.

## 3. Classification of Synaptic Microcircuits in Mushroom-Body Calyx Connecting Projection Neurons with Different Classes of Kenyon Cells

Within all compartments of the MB calyx, neuronal microcircuits are organized by characteristic MG (Figure 1A–C). Each MG comprises a large presynaptic axonal PN bouton surrounded by numerous f-actin rich postsynaptic profiles, most of them originating from KC dendrites [11,12,13,44,45,46,47,48]. In the honeybee and other Hymenoptera, the presynaptic PN boutons and overall shape of the MG are more or less spheroidal (Figure 1B,C), whereas in other insects, including *Drosophila*, MG can be bi-lobed or have more complicated, irregular shapes [48,49,50,51,52]. The regular structure makes quantification of changes in MG densities and numbers most accessible in the honeybee and other Hymenoptera (e.g., by using antibodies with presynaptic proteins) [51,53]. Across different insects, the volume of presynaptic PN boutons in the MB calyx is usually large, and individual boutons comprise very high numbers (up to >60) of presynaptic sites (active zones; see also [13,50]). The centers of PN boutons contain numerous synaptic vesicles and a group of mitochondria, suggesting high energetic costs and a highly dynamic nature of these conspicuously large synaptic complexes [13,36,54,55].

In the following, we largely focus on plastic changes in the PN–KC connectivity within the MG of the MB calyx. However, we want to point out that MB-calyx MG are also targeted by relatively large profiles from a group of recurrent γ-aminobutyric acid (GABA)-ergic MB extrinsic neurons and processes of modulatory extrinsic neurons, particularly octopaminergic and dopaminergic neurons [46,56,57,58,59]. Compared to the numerous, dense synaptic connections between olfactory or visual PNs and KCs, innervation by these modulatory systems is rather sparse and, except for the GABAergic system [46], not yet analyzed at the ultrastructural level in the honeybee.

**Figure 2:**
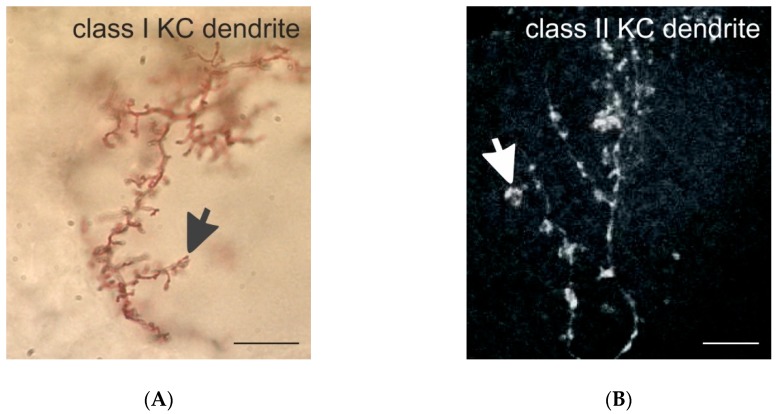
Class I and II KC dendrites in the honeybee. (**A**) Golgi-impregnated dendritic spines of a class I (spiny) KC in the MB-calyx lip. The black arrow marks a dendritic spine. (**B**) Dendritic branches of a class II (clawed) KC in the lip filled with Neurobiotin using iontophoretic injection techniques. The white arrow marks a single dendritic claw. Scale bars in (**A**,**B**) = 10 µm. Images provided by Malu Obermayer in (**A**) and by Kathrin Gehring in (**B**).

In honeybee MBs, KCs can be subdivided into distinct types or classes based on their general dendritic morphology and development (Figure 2A,B). In both the honeybee and the fruit fly, so-called clawed KCs (also termed class II KCs in the honeybee) have been distinguished based on the unique shape of their dendritic branches, their dendritic specializations and the size and localization of the cell bodies (Figure 1A,C and Figure 2B; see also [27,28]). Clawed (class II) KCs arborize over relatively wide regions across the MB calyx in both the honeybee and fruit fly [28,49,60]. Fundamentally different to *Drosophila*, however, a very large number of class I KCs predominantly supply the MB calyx of the honeybee (Figure 1A,B and Figure 2A). These have been termed spiny KCs due to the numerous spine-like protrusions along their dendritic branches [27,28]. Whereas KCs from inside MB-calyx cups (class I KCs) project to both the VL and the ML, KCs outside the MB-calyx cup (class II KCs) do not bifurcate and form a large layer only in the lower-most portion of the VL ([28,44]; for a detailed review see [45]) (see Figure 1 for an example of an outer compact (oc) class II KC, as well as inner compact (ic) and inner non-compact (nc) class I KC). Fahrbach [45] refers to the spiny class I KCs as the typical and most common type of KC in the honeybee (170,000 class I KCs vs only 14,000 class II KCs per MB). Compared to clawed KCs their spiny dendritic trees are more distinctly segregated into and within the three MB-calyx compartments [28]. However, we want to point out here that a systematic, quantitative account of the dendritic branching patterns within and across the two types of KCs is still missing and requires future attention. Earlier investigations have also categorized KCs depending on the position and size of their cell bodies, which relates to the developmental trajectory of the different MB neuroblasts [27,61,62]. The outermost KCs (called outer compact cells) are born first, whereas the innermost ones (inner compact cells) are born last. The cell bodies of the outermost compact cells form a layer around the MB-calyx cup and represent class II or clawed KCs (Figure 1A,C; see also [28]). The spiny class I KCs are arranged within the MB-calyx cup and are classified as non-compact KCs with relatively large somata and dendrites innervating the lip and collar region. The KCs with dendrites in the basal ring compartment have small somata, are located most centrally within the MB-calyx cup and are defined as the inner compact KCs [61]. Axons of both class I and II KCs exit the MB calyx and remain segregated as parallel axonal bundles along the stalk-like pedunculus before forming distinct layers in the medial (ML) and VL, the main output regions of the MBs ([27,28,44]; for a review see [45]). More recent characterizations of KCs according to molecular criteria and gene expression profiles suggest additional subpopulations within the group of class I KCs [63,64,65,66,67].

The differences between the dendritic specializations of spiny and clawed KCs in the honeybee MB calyx most likely have important consequences for their function (Figure 1B,C and Figure 2A,B). Class II KCs form contacts with only a limited number of different PN boutons, whereas spiny KCs may contact very large numbers of individual PN boutons with their numerous dendritic spines ([25,50,60] for *Drosophila*; [13,28] for the honeybee). Our preliminary results in the honeybee suggest that class I KC dendrites may contact individual PN boutons with only one spine, whereas the large claws of class II KCs in *Drosophila* [25,49] form multiple dendritic protrusions with one presynaptic bouton (Figure 1B,C). This difference in the synaptic connectivity and overall dendritic morphology of the two classes of KCs requires further attention and future functional studies, as it most likely has important consequences on the integration and processing of sensory input. We hypothesize that, as clawed KCs contact a relatively small number of PN boutons, they may require input from only a single (or small number of) PN bouton(s) to become depolarized above the threshold; on the other hand, spiny KCs with large dendritic arbors connected to numerous individual PN boutons with only single spines may require highly convergent and coincident inputs from much larger numbers of PN boutons to become stimulated above the threshold and produce action potentials. The functional consequences of these obvious differences in the wiring pattern of class I and II KCs certainly need to be investigated in the future using combinations of ultrastructural, physiological, molecular and modeling approaches (see Section 5).

The qualitative connectome between olfactory PNs and KCs is only partly understood. Whereas co-labeling studies and physiological studies in *Drosophila* suggest a more or less random connectivity between olfactory PNs and KCs [68,69], the axonal projections of olfactory and visual PNs in the MB-calyx lip and collar of the honeybee suggest at least some topographical relationship between the peripheral processing centers (antennal and optic lobes) and representation in the MB calyx (Figure 1A; see also [35,36]). For example, the projections of lateral and medial tract (m- and l-ALT) PNs are at least partially segregated in the olfactory lip [36,70], and the layered organization of visual projections from the medulla and lobula in the collar suggests some degree of segregation of sensory information streams within both the olfactory and visual modalities. A similar layered segregation of projections was found in the basal ring. A distinct region between the lip and collar compartment is occupied by gustatory and potentially mechanosensory projections of neurons transferring sensory information via the subesophageal tract [34,71]. Future work is needed to characterize the qualitative nature of the PN–KC connectome and its flexibility within and across these compartments in the honeybee MB calyx (see Section 5).

## 4. Structural Plasticity of Projection Neuron to Kenyon Cell Connections within Microglomerular Circuits of the Mushroom Body Calyx

The MB calyces undergo substantial volume changes during the adult life of a honeybee (for detailed reviews see [3,53,72]). An early volume increase is most likely caused by an experience-independent internal program, as it also occurs in social isolation, during sensory deprivation and in bees prevented from foraging [73,74,75,76]. Analyses of age-controlled cohorts in both bees and ants have revealed that the MB-calyx volume increase is strongest during early maturation within the first week of adult life (e.g., bees [16]; ants [77]). These early volume increases of MBs have been interpreted as anticipatory plasticity promoting upcoming demanding tasks in behavioral development (bees [74,78,79]; ants [54,80]).

**Figure 3:**
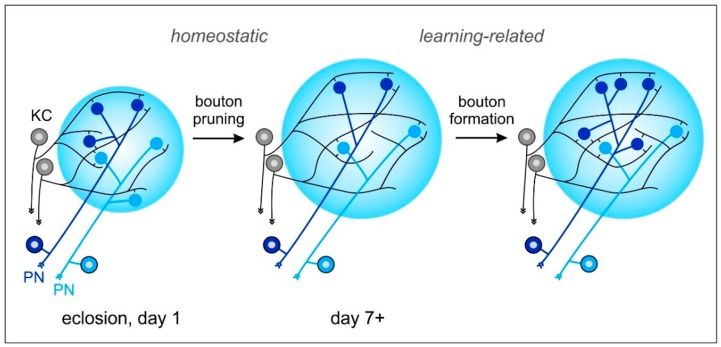
Model of dynamic changes in projection neuron (PN) to Kenyon cell (KC) synaptic connections in the MB-calyx microglomeruli (MG) following non-associative and associative sensory experiences. (Left) Initial number of PN boutons (blue, light blue) in the MB calyx after emergence into adult life. (Middle) Sensory exposure-dependent pruning of PN boutons. At the same time, KC (grey) dendrites expand their network and extend spines to various PN boutons. MG reorganization after increased sensory exposure may be an important preparation of the MB microcircuits for subsequent associative learning and memory formation. (Right) An increase in MG numbers occurs after formation of a transcription-dependent stable long-term memory (LTM) following associative learning. PN: projection neuron; KC: Kenyon cell. See text for further details and references to the original work.

What causes the early volume changes of the MB calyx? Thanks to their spheroidal shape, PN-bouton densities in the MB calyx of the honeybee can be quantified more easily compared to other (non-hymenopteran) insect species using immunolabeling and 3D confocal imaging of synaptic-vesicle associated proteins (e.g., synapsin) that are enriched in the large presynaptic boutons of PNs (Figure 1B,C; see also [3,11,51,53]). Analyses at the cellular level revealed that volume changes in the olfactory and visual compartments of the MB calyx are mainly caused by a massive outgrowth of KC dendrites, which goes along with a decrease in presynaptic PN boutons (pruning) (Figure 3; see also [13,15,16,54,78]). What are the mechanisms driving dendritic KC growth and the changes in densities or numbers of presynaptic PN boutons? Quantitative confocal imaging of PN-bouton densities and estimations of their total numbers has revealed that changes in (non-associative) sensory exposure over the course of their interior–exterior transition cause PN-bouton pruning [15,16,17]. Given that the volume increase of PN boutons is small [13] and that KC dendrites expand massively during the same time period, the results suggest that KC dendritic growth is the main driving force for MB-calyx volume expansion during early maturation and following sensory exposure (Figure 3; [16,78]). Studies by Scholl et al. [15] and Muenz et al. [16] have shown that pruning of PN boutons is associated with first sensory exposure, which is mainly studied in the visual compartments of the MB calyx and by keeping young bees in complete darkness over extended periods of time. Very similar results were found in ants following manipulations of the timing of first visual exposure [54,77,81].

In contrast to PN-bouton pruning following non-associative sensory exposure, associative olfactory learning and the formation of protein-synthesis dependent stable LTM has led to modality-specific and volume-independent increases of PN-bouton densities in olfactory compartments of the MB calyx in the honeybee [18]. A similar effect has also been demonstrated in leaf-cutting ants in regards to the associative (aversive) olfactory learning of odors associated with the formation of an olfactory LTM for detecting unsuitable plant materials [19]. Similarly, in the visual system, experience in naïve *Cataglyphis* desert ants during first learning walks (when ants learn and memorize visual information about panoramic landmarks) has been shown to trigger an increase of MG in the visual compartments (collar) of the MB calyx [20,82,83]. These increases in densities of PN synaptic boutons seen after associative LTM formation suggest the formation of learning-related (Hebbian) structural plasticity in MB-calyx circuits (Figure 3). In contrast to the learning-related increase of PN boutons, pruning of PN boutons upon non-associative (first) sensory exposure most probably represents a form of homeostatic plasticity, adjusting MB input circuits to a drastically changing sensory input during the interior–exterior transition (Figure 3; see also [83]; also reviewed in [20]).

Interestingly, earlier experiments in the honeybee showed that manipulation of early pupal brood care conditions (such as thermoregulatory parameters) resulted in a capacity for changes in the PN–KC connectivity during later stages of the adult life that may be influenced or determined at early phases during postembryonic brood care. Very similar results were also found later in *Camponotus* ants [11,12,84]. Pupal rearing temperature is tightly regulated by thermogenesis in honeybees or brood-carrying behaviors in ants. Deviations from the optimal temperature regimes generated modality-specific effects on synaptic compartments of the MB calyx in freshly emerged adult honeybees and in *Camponotus* ants, whereas rearing under natural temperature regimes led to the highest PN-bouton densities at adult emergence (bees [11]; ants [84]). This represents an interesting case of metaplasticity (for reviews see [85,86]), in the sense that brood-care conditions can have potential consequences for adult behavioral thresholds (e.g., during brood-carrying behavior and division of labor in adult ants [87]). In the honeybee, effects of differences in thermoregulation during pupal stages have been shown to affect sensory processing, learning performance and waggle-dance behavior in adult life, which may be linked to plastic changes in the MBs caused by brood-temperature control [11,88,89,90].

How is the synaptic wiring between PNs and KCs adjusted at the level of individual MG? Three dimensional reconstructions of serial brain sections revealed substantial quantitative changes in the PN–KC connectivity between young nurse bees and experienced foragers using classical electron microscopy (EM) [13]. Individual PN boutons are equipped with up to 70 active zones (AZs; Figure 1B,C), specialized regions for neurotransmitter vesicle release. In honeybee foragers, the numbers of AZs per PN bouton increased in visual PN boutons compared to the situation in young nurse bees, whereas the number of AZs remained similar in olfactory PN boutons in the lip region. In both the olfactory and visual boutons, however, a significant increase in the proportion of ribbon-like to non-ribbon like densities at AZs, as well as in the number of postsynaptic partners per AZ, was found to indicate an increase in synaptic efficiency of individual PN boutons [13]. These findings suggest a ~34% increase in the number of postsynaptic profiles per PN bouton during adult behavioral maturation. This rather drastic increase in the number of postsynaptic profiles attached to individual PN boutons reflects a substantial increase in the synaptic divergence and underlines a remarkable level of structural plasticity in the PN–KC connectome. However, so far, the EM data have only been related to age, and do not allow distinguishing between effects of early maturation, sensory exposure and associative-learning related experience and LTM. The changes observed between young hive bees and experienced foragers most likely reflect the result of a complex combination of these parameters. Therefore, future behavioral manipulations need to be combined with investigations at the ultrastructural level to disentangle non-associative and associative effects of the PN–KC connectivity.

## 5. Open Questions and New Approaches: Towards Understanding the Causes and Consequences of PN–KC Wiring Dynamics in the MB Calyx

### 5.1. Open Questions

MBs represent a highly promising neuronal substrate in the honeybee brain for studying the neuronal mechanisms underlying behavioral plasticity. Although we already have a general idea of the quantitative changes in the PN–KC circuitry resulting from non-associative sensory exposure and learning-related changes (Figure 3), we still lack data on the quantitative wiring dynamics at the level of individual MG under different conditions and according to the qualitative dynamics of the changes in the PN–KC wiring patterns. Furthermore, we need more information on the role of different KC classes, particularly on the question of whether class I (spiny) and II (clawed) KCs differ regarding their roles in PN–KC wiring plasticity. The same is true for different subgroups within class I KCs that differ in morphological and/or molecular criteria. For example, the inner non-compact KCs express CamKII, whereas the inner compact KCs express CREB [63,64]. Both groups of KCs most likely play different roles in memory formation and related changes in PN–KC microcircuits, but might follow different molecular mechanisms. We also lack qualitative data on the wiring specificity between PNs and KCs within sensory modalities (for example in the olfactory system, between l- and m-ALT PNs). This leads to many open questions regarding PN–KC wiring such as: How specific is the match between different KC types and sensory modalities or specialized groups of PNs within modalities? Do some KCs receive convergent inputs from olfactory and visual PNs? How do the two classes of KCs differ regarding their dendritic fields, and how do individual KCs within and across the two classes contribute to the circuitry and plasticity of individual microglomeruli in the MB calyx of freshly emerged bees? How is the PN to KC connectivity in the two subsystems (class I and II KCs), and how is their wiring scheme influenced by experience? How do non-associative and associative sensory experiences shape the connectivity between PN boutons and KC dendrites in visual and olfactory compartments of the MB calyx? Are some KC dendrites recruited only at late stages during adult maturation or LTM formation?

Presently, the olfactory system offers the best access to tackling at least some of these questions as uniglomerular PNs (PNs receiving input from just one antennal lobe glomerulus), and their target region in the MB-calyx lip can be traced (e.g., [91]). The ideal experiment would be to trace PN–KC functional units and track them over time and/or following different manipulations of sensory experience. Based on the concentric organization of the m- and l-ALT PN projections [36], we expect the general PN–KC wiring pattern to be less random in the honeybee compared to the more or less random connectivity suggested for *Drosophila* [69]. The layered organization of visual input in the collar from the optic ganglia points to a similar direction in the honeybee [35]. These considerations suggest that both sensory systems offer some access to the questions of qualitative wiring dynamics. However, as transsynaptic tracers are not yet available for the honeybee, a comprehensive study on experience–dependent changes and dynamics in the qualitative PN–KC connectome is still difficult to pursue.

Quantitative aspects of the PN–KC connectome are easier to address within both the olfactory or visual modalities and across the different KC classes. Class I (inner non-compact and compact spiny) and II (outer compact clawed) KC subpopulations can be selectively back-traced from their output region in the VL (Figure 1A). This can be done for small groups of KCs using systematic iontophoretic injections, electroporation of small numbers of KCs or just manual dye insertion into the different VL layers to study the differential contribution of the two KC classes to individual MG in different MB-calyx compartments and their role in structural plasticity following non-associative and associative experiences. In addition to the PN–KC connectivity, future work on the plasticity of MG microcircuits should also include modulatory innervation such as structural changes in GABAergic feedback neurons [46,92,93] or changes in innervation of the MB calyx by octopamine-, dopamine- and neuropeptidergic systems.

We need better causal connections between individual behavioral performances and different features of plasticity in MB-calyx microcircuits. This requires sophisticated behavioral experiments, for example by building on manipulations performed in earlier experiments on LTM formation and by combining those with pharmacological inhibition of protein synthesis or knockdown of protein expression [18,94]. Based on previous studies on individual olfactory learning success in honeybees [95,96] and its correlation with physiological plasticity [23,95], this direction appears very promising, especially in light of recent manipulations of sensory experience [17]. However, the critical point still is how we can obtain true causal relationships. While examining this question we have to keep in mind that although the high level of structural plasticity in the MBs is highly suggestive for an important function in behavioral plasticity, the MB calyx may not be the only site expressing long-term structural plasticity in behaviorally relevant neurocircuits. Recent results suggest that structural plasticity may be more distributed than previously thought. For example, results of visual (color) learning in ants have revealed plasticity in the MBs, optic lobes and central complex [82,97,98]. Furthermore, recent results in *Drosophila* indicate that LTM may also form independently of protein synthesis in the lateral horn (LH) [99]. Future neurophysiological recordings from MB output neurons (MBs; see below) and by tracing of their modulatory connections to other brain regions will be important towards understanding the functional (behavioral) consequences of changes in MB circuits.

### 5.2. New Approaches and Methodological Advances

Future studies on plasticity in MBs certainly will benefit from new methodological developments. Investigations at both the light and electron microscopic levels combined with sophisticated behavioral manipulations and physiological studies (electrophysiology and live-imaging) will allow us to address several of the questions highlighted above, such as how to disentangle differential effects of non-associative and associative experience of the PN–KC wiring patterns in different sensory compartments and for different classes of KCs in the MB calyx.

How do external and internal factors affect the PN–KC connectome? New neuroanatomical techniques will help to identify underlying plastic changes in MB microcircuits. Serial block-face EM techniques have already started to promote connectome studies [100] and will be important for circuit analyses in the MB calyx of the honeybee, especially when combined with the tracing of individual neurons. To improve the identification of quantitative changes in MB-calyx MG, we recently succeeded in combining immunolabeling of synaptic antibodies with f-actin-phalloidin staining in whole brain preparations, which allows confocal microscopy volume measurements of the entire MB calyces to be combined with quantifications of PN-bouton densities and numbers [20,52,83]. However, methods for automated quantification of MB-calyx MG in most cases has turned out to be insufficient for counting MG numbers in large tissue volumes or the entire MB calyx [51]. This problem might be solved in the near future by combining pre- and postsynaptic labeling with deep tissue imaging using multiphoton microscopy, improved image processing tools (e.g., deconvolution) and/or combined with intelligent image processing using flexible MG detection algorithms [101].

On the side of tissue processing, the recently developed expansion microscopy offers a promising opportunity for quantifying changes at the level of individual MG, especially when combined with superresolution light microscopy tools (e.g., [102,103,104]). These techniques allow details below 100 nm to be resolved down to the level of individual AZs, which opens up possibilities for quantitative 3D imaging of tissue volumes without the need for tedious serial EM sectioning. To identify changes at even higher detail and molecular levels, new advanced EM techniques such as EM tomography or correlative EM techniques (array tomography) combined with superresolution microscopy can allow fluorescent labeling at the single molecule level, along with EM resolution of structural details [55]. The new microscopic techniques will greatly facilitate the discovery of mechanisms underlying changes in synaptic connectivity at the level of individual MG.

How do changes in the PN–KC connectivity following non-associative or associative experience affect the neuronal representation of sensory input? Improvements in neurophysiological tools, especially the long-term recording of MB output neurons (MBONs), provide an important bridge between structure, function and behavior. The combination of behavioral manipulations, neuroanatomical circuit analyses and neurophysiology is particularly promising. The refinement of long-term multi-unit recording techniques has been an important step in this direction [105] and has led to simultaneous recordings of groups of neurons at multiple sites (neuropils) within the brain [106,107,108]. Multi-unit recordings over long time periods offer the advantage of analyzing functional consequences of structural synaptic plasticity in MB microcircuits. Recent studies on MBONs (receiving convergent inputs from many KCs) have revealed important insights on information processing properties of MB such as multisensory convergence, temporal coding properties, learning-induced changes in MBON activity and stimulus categorization by multisensory MBONs [106,109,110]. Intracellular recordings from individual neurons within a specific group of GABAergic feedback neurons (A3 PCT neurons, a specific group of MBONs forming recurrent circuits from the MB lobes to the MB-calyx input) has highlighted the role of these neurons in mediating memory-related changes from the MB output to the MB-calyx input [22,58,59,93,111]. Physiological recordings from KCs, so far, have been mostly limited to the calcium imaging of spatial activation patterns, which has revealed important insights into experience-related changes in spatial activation patterns of pre- and postsynaptic elements in MB-calyx MG [21,23,112]. In situ electrophysiological recordings from KCs have shown that their highly phasic (sparse) temporal coding properties are most probably caused by intrinsic ion channel properties [113,114]. However, in situ patch-clamp recordings from individual KC somata are extremely difficult to obtain in the honeybee, mostly due to their small size and limited accessibility. In future attempts, application of multiphoton live-imaging combined with novel voltage-sensitive dyes might open up access to both the spatial and temporal resolution needed to address the consequences of plastic changes in different KC classes following neuromodulatory influences [115].

What is the genetic and molecular basis of plastic changes in MB microcircuits? Genetic and molecular manipulations are important tools for addressing causal relationships between neuronal and behavioral plasticity. Transcriptomic approaches or analyses of immediate early gene (IEG) expression have revealed interesting candidate genes and gene activation patterns relevant to brain plasticity [116,117]. Monitoring the spatial expression patterns of IEGs using RNA in situ hybridization or immunolabeling of IEG products may be extremely helpful in the future to identify neuronal substrates in the brain involved in structural neuronal plasticity in a specific behavioral context [118,119,120]. The crucial part, however, will be genetic manipulation experiments using knockdown or knockout techniques such as RNA interference or CRISPR/Cas9 gene editing. Although CRISPR/Cas9 and related approaches have recently emerged as a promising tool, including for the honeybee [67,121,122,123], candidate genes involved in adult neuronal plasticity most probably play equally important roles during development, and knockout of these genes, if not compensated by other genes, will lead to early death or severe developmental defects. Furthermore, the CRISPR/Cas9 technique is difficult to apply and is time-consuming in the honeybee, mainly due to the reproductive biology and social lifestyle of honeybee colonies [67]. Knockdown manipulations using RNA interference have been a successful alternative in some cases. For example, a recent study using brain RNAi demonstrated the role of CamKII in early and late olfactory LTM formation [94]. However, brain RNAi injections allow only a limited resolution of target regions and, in other cases, the use of RNAi in the adult honeybee brain has failed. In conclusion, genetic manipulation is still difficult to apply in the honeybee, and we need refined or completely novel techniques in the future that ideally would allow conditional knockouts of target genes in adult bees.

Can modeling approaches help us to understand the functional role of changes in MB microcircuits? The use of anatomical, physiological and behavioral data for computational modeling approaches is a highly promising line for future research. For example, a recent modeling study revealed the importance of structural plasticity in MB input synapses for certain aspects in complex learning [124], and the combination of modeling with physiological experiments highlighted the role of structural plasticity in MB-calyx microcircuits nicely [23]. Our growing knowledge on the structure of the circuits, their dynamics in connectivity and the different levels of plasticity in MB circuits opens new terrain for future modeling studies that will greatly help us to test model predictions using clever combinations of behavioral, anatomical and physiological manipulation experiments.

What are the evolutionary foundations of the high levels of neuroplasticity in the honeybee? The evolutionary foundations of the enhanced levels of neuroplasticity in the honeybee and other social insects still remain unclear both at the cellular and molecular levels. We need more comparative approaches in the future, preferentially looking into closely related social and solitary species [31,32]—or facultatively social insect species. The comparative approach will be very elusive at the molecular level, such as in identifying the genes or epigenetic mechanisms underlying the evolution of increased levels of developmental and adult plasticity in social-insect brains, which is similar to what was shown earlier in cases of the epigenetic mechanisms underlying female caste determination and the regulation of ovary development in honeybees via nutrition-related changes in DNA methylation [125].

Considering the multitude of possible future approaches and the diversity of individual behaviors relevant for social organization, future studies on the honeybees offer highly promising integrative approaches for the role of neuroplasticity in controlling behavior in a social context. The studies reviewed here revealed astonishing levels of developmental and adult plasticity in neuronal circuits and the related processing of sensory information in the honeybee brain. High levels of neuroplasticity promote behavioral flexibility—a highly relevant feature of honeybee sociality. The high degree of structural plasticity in numerous parallel MB-calyx microcircuits will continue to be a unique system for addressing causal relationships between neuronal and behavioral plasticity, all the way up to their impact on bee sociality at the colony level.

## Abbreviations

AIOT	anterior inferior optic tract
ASOT	anterior superior optic tract
AL	antennal lobe
AZ	active zone of synapse
br	basal ring of the MB calyx
CamKII	Ca2+/calmodulin-dependent protein kinase II
co	MB calyx collar
CREB	cAMP response element-binding protein
ic	inner compact KCs
CB	central body
IEG	immediate early genes
KC	Kenyon cell
LA	lamina
l-ALT	lateral antennal lobe tract
LCA	lateral calyx
li	MB calyx lip
LH	lateral horn
LO	lobula
LOT	lobular tract
LTM	long-term memory
m-ALT	medial antennal lobe tract
MCA	medial calyx
MB	mushroom body
MBON	MB output neuron
ME	medulla
MG	microglomerulus, microglomeruli
ML	medial lobe of the MB
nc	non-compact KCs
oc	outer compact KCs
PED	peduncle of the MB
PN	projection neuron
RET	retina
RNAi	RNA interference
VL	vertical lobe of the MB
